# Factors affecting satisfaction on antenatal care services in Sarawak, Malaysia: evidence from a cross sectional study

**DOI:** 10.1186/s40064-016-2447-3

**Published:** 2016-06-16

**Authors:** Md Mizanur Rahman, Deburra Peak Ngadan, Mohammad Taha Arif

**Affiliations:** Department of Community Medicine and Public Health, Faculty of Medicine and Health Sciences, Universiti Malaysia Sarawak, 94300 Kota Samarahan, Sarawak Malaysia

**Keywords:** Antenatal care, Level of satisfaction, Sarawak

## Abstract

**Background:**

High levels of satisfaction among women with the antenatal care services will increase the compliance of antenatal visits during pregnancy. Thus, this study was done to assess the level of satisfaction among women on the quality of antenatal care received and the factors influencing thereof.

**Methods:**

This was a cross-sectional study conducted in the three zones of Sarawak. Women aged 18 years and above, irrespective of ethnic groups, having children aged 3 years and below were included in the study. Data was collected by face-to-face interview using interview schedule. A validated Patient Satisfaction Questionnaire (PSQ-18) was used to assess the satisfaction with antenatal care. A total of 1236 data was analysed using IBM SPSS version 22.0. A p value <0.05 was considered as statistically significant.

**Results:**

A multinomial logistic regression analysis revealed that Bidayuh 17.4 % was less likely to be highly satisfied with antenatal care. Similarly, respondents with secondary level of education 29.9 % were less likely to be highly satisfied, whereas, respondents having primary level of education, 1.6 % were less likely to be highly satisfied. However, those who did not spend any money as out of pocket expenses were 1.935 times more likely to be highly satisfied with antenatal care.

**Conclusion:**

Overall the studied women were satisfied with the antenatal care services. Ethnicity, level of education and out of pocket expenses appeared to be important predictors of satisfaction with antenatal care. The finding recommends the community-based and language-specific interventions should be implemented to sustain the satisfaction of maternal care.

## Background

Utilization or antenatal visit has been shown to improve birth outcome as well as the maternal outcome during pregnancy-related events, giving a positive impact when the visit frequency and care are adequate (Garrido 2009). The World Health Organization (WHO) has recommended a minimum of four (4) antenatal visits per pregnancy for an optimal birth outcome and to reduce maternal risks per pregnancy, especially, for developing countries (AbouZahr and Wardlaw 2003). Satisfaction has been one of the outcome measures of quality of care as well (Koblinsky et al. 1999). Satisfaction studies on maternal health services, particularly antenatal service have been carried out as a measurement of outcomes of public health policies. It is also an indirect reflection of the quality of antenatal care service provided to the patients (Hasan et al. 2007), their experience and their role as end-users in the health care system, such as the satisfaction study done in National University Hospital, Kuala Lumpur (Pitaloka and Rizal 2006) and in community-based study in Vietnam (Tran et al. 2012). Health care provider factors such as private care versus government clinics have also been studied showing a higher satisfaction in the private care (Nketiah-Amponsah 2009; Zeine et al. 2010), caregivers’ positive attitude (Zeine et al. 2010; Hildingsson and Radestad 2004; Esimai and Omoniyi-Esan 2009; Ghobashi and Khandekar 2008; Davey et al. 2005), continuity of care (Nketiah-Amponsah 2009; Davey et al. 2005; Biró et al. 2003; Fan et al. 2005), distance (Nketiah-Amponsah 2009) and short waiting time (Esimai and Omoniyi-Esan 2009), but long duration with care givers (Ghobashi and Khandekar 2008; Davey et al. 2005), have been shown to increase the satisfaction of patients receiving antenatal care services. Other than the health care providers aspect, maternal age has been shown to affect the satisfaction of antenatal service, with young mothers aged less than 25 years (Hasan et al. 2007) being more satisfied while some studies showed that older mothers aged more than 30 years (Zeine et al. 2010; Yohannes et al. 2013) were more satisfied with the services and were factors associated with utilization of service (Zeine et al. 2010). The level of education is another determining factor influencing satisfaction with antenatal care services (Hasan et al. 2007; Nketiah-Amponsah 2009; Zeine et al. 2010; Yohannes et al. 2013). Other family related factors are family size (Zeine et al. 2010), household income and costs per antenatal visit (Zeine et al. 2010; Overbosch et al. 2004). In developed countries such as in Sweden (Hildingsson and Radestad 2004) and developing countries such as Vietnam (Tran et al. 2012) and Malaysia (Pitaloka and Rizal 2006), the frequency of antenatal visit itself was the determinants of satisfaction, whereby higher numbers of visit increased the satisfaction of antenatal services. The significance of this study towards the current knowledge of antenatal care services was in identifying the different domains of antenatal service provision in which the women were satisfied with; as well as identifying the factors affecting the level of satisfaction among the women, which would increase the compliance and usage of maternity services. Thus, in this context, the main objective of this study was to determine the factors associated with the level of satisfaction among women visiting the Mother and Child Health clinics in Sarawak during their last pregnancy.

## Methods

### Study design and sampling procedure

This was a cross-sectional study conducted in three (3) different divisions of the eleven divisions in Sarawak. Every three (3) out of five (5) villages were selected through the Mother and Child Health Clinic registry. A multi-stage cluster random sampling was used for sampling procedure. All households of the villages with mothers having the youngest child of 3 years and below were visited. All respondents who did not consent or unwilling to participate; aged below 18 years; non-Malaysian citizenship; incapable of answering the questionnaires were excluded from the study. A total of 1400 households were selected initially, however, a total of 1236 women gave their consent for participation.

### Instrument development and data collection procedure

A modified data collection instrument was developed based on Demographic Health Survey (DHS) (Pitaloka and Rizal 2006), PSQ-18, (Marshall and Hays 1994) and others relevant instruments (Sarawak State Health Department 2003; Rahman 1997). Data collection was done using an interviewer-administered questionnaire. The questionnaire consisted of four main parts, which were (1) socio-demographic characteristics and the variables were maternal age, ethnicity, level of education, occupation, household income and family size. (2) Antenatal care history, and the variables were number of antenatal visits, gestational age at booking, antenatal attendant, time required to reach the clinic and out of pocket expenses, (3) delivery care history and (4) postnatal care history. For each maternity care services, patient satisfaction questions were asked regarding the services received. The level of satisfaction with antenatal care was determined by seven domains of satisfaction. Each domain has Likert’s scale questions. After summation of all domains score, it was checked for normality. The satisfaction score was divided into quartiles. The lowest quartile as poor and middle two quartiles as average and the last quartile categorized as highly satisfied with antenatal care. In the current analysis, only antenatal care history was considered. Before field operation, a pre-test of the questionnaire was done in a non-sample area with the translated National language (Bahasa Malaysia) questionnaire. A minor change was made following pre-test of the questionnaire.

### Ethical considerations

The study proposal was approved by the Technical Review Committee of the Faculty of Medicine and Health Sciences, Universiti Malaysia Sarawak (UNIMAS) and the National Medical Research Registry (NMRR). Ethical clearance was also obtained from the Institutional Review Board of the Faculty of Medicine and Health Sciences and Institute of Public Health, Malaysia. Informed written consent was obtained from the respondents before data collection. The respondents were also assured of data confidentiality.

### Data processing and analysis

After data collection, the data were checked manually for completeness and inconsistencies. Data entry and analysis was by IBM SPSS version 22.0 (Corp 2013). Missing data were carefully examined and was imputed by SPSS. After validation, descriptive analysis was done and descriptive statistics were presented in frequency tables. A multinomial logistic regression analysis was done to identify factors affecting the level of satisfaction with antenatal care (Tabachnick and Fidell 2013). A p value <0.05 was considered as statistically significant.

## Results

### Socio-demographic characteristics

The mean (SD) age was 28.3 (6.1) years ranging from 18 to 49 years. The highest percentage of them were Iban (40 %) followed by Bidayuh (30.3 %) and Malay (17.7 %). More than two-thirds (69.5 %) were Christian followed by Muslim (27.3 %). The highest percentage of respondents had secondary level of education (69.7 %) followed by higher secondary and above (16.9 %). The majority of them were housewives (76.9 %) and the rest were engaged in service or other jobs. The median income was MYR 1000 and the average family size was 5.9 (Table 1).

**Table 1:** Socio-demographic characteristics of the respondents (n = 1236)

Characteristics	Frequency	%	95 % CI	
			Lower bound	Upper bound
*Age in years*				
<20	77	6.2	4.9	7.5
20–29	667	54.0	51.1	56.9
30–39	438	35.4	32.6	38.1
40–49	54	4.4	3.3	5.5
Mean (SD) years	28.3 (6.1)		28.0	28.7
*Ethnicity*				
Malay	219	17.7	15.5	19.8
Iban	494	40.0	37.1	42.6
Bidayuh	375	30.3	27.9	32.8
Others (e.g. Chinese etc.)	148	12.0	10.2	13.8
*Religion*				
Christian	859	69.5	67.0	72.0
Muslim	337	27.3	24.8	29.8
Others	40	3.2	2.3	4.3
*Level of education*				
No formal education	25	2.0	1.3	2.8
Primary	141	11.4	9.6	13.2
Secondary	861	69.7	67.1	72.1
Higher secondary and above	209	16.9	14.9	19.0
*Occupation*				
Housewife	951	76.9	74.4	79.2
Government	132	10.7	8.9	12.5
Private	109	8.8	7.3	10.4
Self employed	44	3.6	2.6	4.6
*Household income (MYR)*				
<1000	586	47.4	44.7	50.5
1000–1999	356	28.8	26.4	31.4
≥2000	294	23.8	21.4	26.1
Median	1000.00		950.00	1000.00
*Family size*				
2–3	170	13.8	11.8	15.7
4–5	467	37.8	34.9	40.6
6–7	334	27.0	24.4	29.6
≥8	265	21.4	19.2	23.7
Mean (SD)	5.9 (2.5)		5.8	6.0

*CI* confidence interval

### Characteristics of antenatal care

The majority of the respondents made more than nine antenatal visit during their last pregnancy (72.2 %) followed by 5–8 times (20.8 %) and only 7 % had 1–4 times antenatal visits. Three-fifths (60.9 %) of them booked antenatal visit before the third month followed by another one-third (32.4 %) who booked between 3 and 5 months of gestation. Half of them were attended by a nurse (50.9 %). However, two-fifths (38.1 %) were attended by both doctor and nurse. On an average 18 min were required to attend the clinic. About three-fifths (57.5 %) were attended to within 15 min. It was reported that one-fifth (18.9 %) did not have any out of pocket expenses. However, about half (48.7 %) had spent 11–50 ringgit per visit (Table 2).

**Table 2:** Percentage distribution of respondents by the number of antenatal visits, gestational age at booking, place of booking and out of pocket expenses (n = 1236)

Characteristics	Frequency	%	95 % CI	
			Lower limit	Upper limit
*No. of antenatal visits*				
1–4	86	7.0	5.7	8.4
5–8	257	20.8	18.4	23.0
≥9	893	72.2	69.8	74.8
Mean (SD)	9.7 (3.6)		9.51	9.90
*Gestational age at booking (months)*				
<3	753	60.9	58.3	63.8
3–5	400	32.4	29.8	35.0
≥6	83	6.7	5.3	8.2
*Antenatal attendant*				
Doctor	136	11.0	9.2	12.6
Nurse	629	50.9	48.2	53.8
Both	471	38.1	35.5	40.7
*Time required to nearest MCH Clinic (min)*				
<15	711	57.5	54.8	60.3
15–29	428	34.6	31.9	37.1
≥30	97	7.8	6.2	9.4
Mean (SD) min	18.05 min			
*Expenses per antenatal visit (MYR)*				
None	233	18.9	16.7	21.1
1–10	270	21.8	19.6	24.3
11–50	602	48.7	46.0	51.6
≥51	131	10.6	8.9	12.4

*CI* confidence interval

### Factors affecting satisfaction on antenatal care: multinomial logistic regression analysis

A multinomial logistic regression was done to examine the factors affecting the level of satisfaction with antenatal care in which the satisfaction score was divided into three groups based on quartile score (poor, average and highly satisfied). It was found that 24.6 % were poorly satisfied and considered as reference category and 51 % had the average satisfaction and another 24.6 % were highly satisfied with antenatal care. Initially, all the explanatory variables were analyzed with the level of satisfaction using Chi square test of independence. The variables that were statistically significant in Chi square test were entered into mutinomial logistic regression model (Tabachnick and Fidell 2013). In the final model, ethnic group, level of education, and out of pocket expenses appeared to be important predictors of the level of satisfaction with antenatal care (p < 0.05). The full model showed that it was statistically significant χ^2^(24, 1211) = 50.014; p < 0.001 indicating that the model was able to distinguish between respondents who were satisfied and unsatisfied with the antenatal care. This model contains the four independent variables explained between 4.0 % (Cox and Snell R square) and 4.6 % (Nagelkerke R^2^) of the variance in the level of satisfaction. It was also able to classify 51.4 % of the cases. The goodness of fit indices was not statistically significant [Chi square (df) = 269.042(256); p > 0.05] which indicated good fitted model.

Analysis indicated that Bidayuh 17.4 % were less likely to be highly satisfied with antenatal care. Similarly, respondents with secondary level of education 29.9 % were less likely to be highly satisfied, whereas, respondents having primary level of education, 1.6 % were less likely to be highly satisfied. Regarding, out of pocket expenses, those who did not spend any money were 1.935 times more likely to be highly satisfied with antenatal care (Table 3).

**Table 3:** Factors affecting satisfaction on antenatal care: multinomial logistic regression analysis

Characteristics	Average satisfaction				Highly satisfied			
	β	Adj. OR	95 % CI		β	Adj. OR	95 % CI	
			Lower bound	Upper bound			Lower bound	Upper bound
*Ethnicity*								
Malay	0.005	1.005	0.564	1.789	0.062	1.064	0.565	2.005
Iban	−0.148	0.862	0.524	1.42	−0.279	0.757	0.433	1.32
Bidayuh	−0.306	0.736	0.443	1.223	−0.826**	0.438	0.244	0.785
Others (RC)		1				1		
*Level of education*								
No formal education	−0.788	0.455	0.158	1.31	−1.029	0.357	0.106	1.204
Primary	−0.484	0.616	0.327	1.161	−0.984**	0.374	0.182	0.769
Secondary	−0.334	0.716	0.441	1.164	−0.701**	0.496	0.291	0.845
Higher secondary and above (RC)		1				1		
*Occupation*								
Housewife	0.088	1.091	0.476	2.503	0.085	1.089	0.428	2.772
Government	−0.127	0.881	0.338	2.293	−0.251	0.778	0.265	2.282
Private	−0.845	0.43	0.17	1.087	−0.539	0.583	0.205	1.658
Self-employed (RC)		1				1		
*Expenses for ANC visit (RM)*								
None	0.478	1.613	0.936	2.778	0.660*	1.935	1.028	3.643
1–10	0.325	1.384	0.821	2.335	0.494	1.639	0.888	3.023
11–50	0.308	1.361	0.857	2.161	0.145	1.156	0.664	2.015
≥51 (RC)		1				1		
Constant	0.921	0.088			0.682	0.259		

Dependent variable: level of satisfaction (poor as reference, average and highly satisfied)

Model Chi square (df, n) = 50.014 (24, 1211); p < 0.001

Goodness of fit [χ^2^(df) = 269.042(256); p = 0.276]

* p < 0.03; ** p < 0.01; *** p < 0.001

## Discussion

The maternal age in this study was highest in the age range of 20–29 years old, with the mean age of 28.3 years, showing a younger age group. A similar result was found in a study in Kuala Lumpur with a median of 29 years (Pitaloka and Rizal 2006). Peculiar to this study was the ethnic makeup, with two-thirds were from the indigenous races of Sarawak known also as Dayak, compared to studies done in Peninsula Malaysia with the majority of Malays, Chinese and Indians (Pitaloka and Rizal 2006). Minimal tertiary level education and housewives show similar findings as found in other countries with a rural setting such as in Bangladesh (Hasan et al. 2007), Egypt (Montasser et al. 2012), Ethiopia (Zeine et al. 2010), India (Khanam et al. 2012) and Asian countries such as Vietnam (Tran et al. 2012). Even though the highest income group was below the recommended minimal monthly income of MYR 850, the mean was MYR 143.04 and the median was MYR 1000 showing the patterns of rural socioeconomic status. However, this was lower as compared to the national census of mean household income of RM 3080 in rural Malaysia (Department of Statistics, Malaysia 2012). The median family size was 5, with an average of four children. This was in accordance with the declining fertility rate, which was encountered by the researcher during community survey as well as findings from the literature regarding reducing the fertility rate in Malaysia (Yadav 2012).

The characteristics of antenatal care received during last pregnancy revealed that the majority of them visited, at least, the state-recommended total of eight visits per pregnancy, which was similar to a high number of antenatal visits in developed countries such as in the United Kingdom (Redshaw and Heikkila 2010) and Sweden (Hildingsson et al. 2002). Two-thirds of the women visited the MCH clinic before 3 months of gestational age, which was also similar to Vietnam (Tran et al. 2012) and United Kingdom (Redshaw and Heikkila 2010). The median distance to the nearest MCH clinic was 15 min, closer distance compared to Bangladesh (Hasan et al. 2007), but similar to Tanzania whereby the nearest clinic was located in the village itself (Rockers et al. 2009). Understandably, more than three-quarters of the respondents used their own transport for follow-up; with the median amount of MYR 37.00 per visit.

The level of satisfaction with antenatal care services in this study was high, similar to other studies done in Kuala Lumpur, Malaysia (Pitaloka and Rizal 2006) Oman (Ghobashi and Khandekar 2008), Bangladesh (Hasan et al. 2007), United Kingdom (Redshaw and Heikkila 2010), Ethiopia (Esimai and Omoniyi-Esan 2009) and Scotland (Teijlingen et al. 2003). Even though they were satisfied with the waiting hours, however, they were less satisfied with the duration spent during consultation and treatment with medical staff, similar to findings in an urban antenatal clinic setting in Kuala Lumpur (Pitaloka and Rizal 2006) and in developed countries such as Oman (Ghobashi and Khandekar 2008).

Ethnicity, level of education, and out of pocket expenses appeared to be important factors for satisfaction on antenatal care. The studies done in developing countries such as in Nigeria and Oman also showed similar findings of race affecting the level of satisfaction on antenatal care services (Ghobashi and Khandekar 2008; Oladapo et al. 2008). Travailing far to the nearest clinic has been shown to reduce the level of satisfaction found in previous studies in Ghana (Overbosch et al. 2004) and Vietnam (Tran et al. 2012). There have been mixed findings regarding the level of education affecting satisfaction among women in antenatal care services. The lower level of education was associated with dissatisfaction such as in Sweden (Hildingsson and Radestad 2004), whereas tertiary and higher education were associated with high satisfaction levels as found in Nigeria (Esimai and Omoniyi-Esan 2009) and Ethiopia (Yohannes et al. 2013).

This study identified several limitations such as the cross-sectional design of this study and the dependence on recall memory of the respondents. The strength is its state-wide implementation where the respondents were recruited mainly from villages in the suburban and rural communities. This allows the findings to represent the community in these areas of Sarawak.

## Conclusion

Overall, women were satisfied with the antenatal care services provided in the maternal and child health clinics in Sarawak. The finding that the diversified cultural society in Sarawak showed disparity in the level of satisfaction with antenatal care needs further exploration. The finding suggests that health education and public health program interventions should be more focused to local settings, which are community-oriented and culturally suited language-specific approach, and not a ‘one size fit all’ approach for maternal health-related programs. Current “no-fee charges” for antenatal care services in Malaysia should be maintained, as the mother still prefer free services.
